# Beyond the apnea–hypopnea index in COPD–OSA overlap syndrome: a mechanism-informed framework for nocturnal oxygenation and ventilation

**DOI:** 10.3389/fphys.2026.1889633

**Published:** 2026-08-13

**Authors:** Xize Zhang, Xue Han, Shaodan Hu, Li Shi

**Affiliations:** 1 College of Traditional Chinese Medicine, Changchun University of Chinese Medicine, Changchun, Jilin, China; 2 Department of Pulmonary Disease, Affiliated Hospital of Changchun University of Chinese Medicine, Changchun, Jilin, China

**Keywords:** COPD-OSA overlap syndrome, hypoxic burden, nocturnal hypoxemia, positive airway pressure, sleep-related hypoventilation, transcutaneous carbon dioxide

## Abstract

**Background:**

In chronic obstructive pulmonary disease (COPD)–obstructive sleep apnea (OSA) overlap syndrome, the apnea–hypopnea index (AHI) confirms and grades OSA but does not fully explain nocturnal hypoxemia or carbon dioxide retention.

**Objective:**

We propose a post-diagnostic, mechanism-informed framework for interpreting nocturnal oxygenation and ventilation after both COPD and OSA have been objectively confirmed.

**Methods:**

This targeted narrative review synthesized PubMed literature and major guideline sources through 20 May 2026. Reporting followed established narrative-review quality principles, and evidence use was categorized as guideline-supported, direct overlap-syndrome observational evidence, indirect COPD or OSA evidence, or physiological inference.

**Results:**

The framework separates three baseline mechanisms: event-related upper-airway obstruction, sustained low-baseline oxygenation or gas-exchange impairment, and sleep-related hypoventilation or chronic hypercapnia. Residual hypoxemia after optimized positive airway pressure or noninvasive ventilation is treated as a post-treatment reassessment scenario rather than a baseline phenotype. Suggested core reporting includes the AHI measurement method, hypopnea rule, oxygen desaturation threshold, time spent below specified oxygen saturation thresholds, mean and verified minimum oxygen saturation, rapid eye movement and supine exposure, treatment context, oximetry settings, and carbon dioxide monitoring when indicated.

**Conclusion:**

AHI remains essential for diagnosing OSA, but oxygenation and ventilation in overlap syndrome may be better interpreted by mechanism. The proposed framework is an interpretive and reporting scaffold rather than a treatment algorithm, and its clinical utility, reproducibility, and prognostic value require prospective validation in well-characterized cohorts.

## Introduction

Chronic obstructive pulmonary disease (COPD) and obstructive sleep apnea (OSA) can coexist as COPD–OSA overlap syndrome, a clinically important interaction associated with adverse respiratory and cardiovascular outcomes (McNicholas, 2017; McNicholas et al., 2013; Chaouat et al., 1995; Marin et al., 2010; Shawon et al., 2017; Malhotra et al., 2018). The apnea–hypopnea index (AHI) remains necessary for confirming and grading OSA, but it does not fully describe the nocturnal oxygenation and ventilatory burden created by coexisting COPD. A patient with a modest AHI may spend prolonged periods at low oxygen saturation because reduced pulmonary reserve, ventilation–perfusion mismatch, or sleep-related hypoventilation limits recovery, whereas another patient with a high AHI may show predominantly intermittent desaturation with relatively preserved between-event oxygenation. AHI also does not directly capture sustained low-baseline oxygenation, desaturation depth and duration, delayed reoxygenation, or carbon dioxide retention. This discordance between event frequency, cumulative hypoxemic exposure, and ventilatory adequacy is the central physiological problem addressed in this Review.

Existing reviews have comprehensively summarized the definition, epidemiology, screening, pathophysiology, cardiovascular risk, biomarkers, and management of overlap syndrome, including positive airway pressure, noninvasive ventilation, oxygen therapy, pulmonary rehabilitation, and integrated care (Owens and Malhotra, 2010; McNicholas, 2009; Budhiraja et al., 2015; Wei et al., 2026; Voulgaris et al., 2026). However, less attention has been given to the post-diagnostic question that arises after COPD and OSA have both been objectively confirmed: what mechanism is primarily responsible for nocturnal hypoxemia or carbon dioxide retention in an individual patient? Nocturnal desaturation in COPD without diagnostic OSA represents isolated nocturnal hypoxemia rather than overlap syndrome, and the same oxygen saturation pattern may reflect recurrent upper-airway obstruction, limited gas-exchange reserve, sleep-related hypoventilation, or a combination of these processes. A structured approach is therefore needed to separate diagnostic confirmation from physiological interpretation and to distinguish baseline mechanisms from abnormalities that persist only after treatment.

This targeted narrative review proposes a post-diagnostic, mechanism-informed framework organized around three non-exclusive baseline mechanisms: event-related upper-airway obstruction, sustained low-baseline oxygenation or gas-exchange impairment, and sleep-related hypoventilation or chronic hypercapnia. Residual hypoxemia after optimized positive airway pressure or noninvasive ventilation is considered separately as a post-treatment reassessment scenario rather than a baseline phenotype. The Review also distinguishes core from extended reporting items and classifies supporting evidence as guideline-supported, direct overlap-syndrome observational evidence, indirect COPD or OSA evidence, or physiological inference. Its intended contribution is not another broad management summary, but a structured and testable approach to interpreting nocturnal oxygenation and ventilation once both component diseases have been confirmed. The framework should be viewed as an interpretive and reporting scaffold rather than a treatment algorithm or guideline-level recommendation; its clinical utility, reproducibility, and prognostic value require prospective evaluation in well-characterized cohorts.

## Methods: review approach and evidence classification

This targeted narrative review was designed for conceptual, mechanism-oriented clinical synthesis rather than effect estimation. It was not intended for exhaustive study capture, duplicate screening, formal risk-of-bias assessment, certainty grading, pooled estimation, or evidence mapping. Reporting was guided by SANRA principles for clarity, rationale, search transparency, and balanced evidence presentation (Baethge et al., 2019).

We searched PubMed/MEDLINE for English-language adult literature from database inception to 27 April 2026, with a top-up search performed through 20 May 2026. Search terms covered COPD, OSA, overlap syndrome, nocturnal hypoxemia/desaturation, hypoventilation/hypercapnia, time spent with oxygen saturation below 90% or 88% (T90/T88), oxygen desaturation index (ODI), hypoxic burden, transcutaneous carbon dioxide (TcCO_2_), continuous positive airway pressure (CPAP), noninvasive ventilation (NIV), and oxygen therapy. Guideline sources included the Global Initiative for Chronic Obstructive Lung Disease (GOLD), American Academy of Sleep Medicine (AASM) diagnostic/scoring guidance, NICE NG202, American Thoracic Society (ATS) guidance on obesity hypoventilation syndrome (OHS), ATS/European Respiratory Society (ERS) guidance on long-term NIV in chronic hypercapnic COPD, and ATS guidance on home oxygen therapy. **Supplementary Material 1** provides the complete search syntax, guideline sources, hand-search strategy, selection priorities, and SANRA-based checklist.

Records were selected purposively for four prespecified domains: objective confirmation of COPD and OSA; nocturnal oxygenation burden; ventilatory adequacy and CO_2_ retention; and responses to CPAP, NIV, oxygen, and COPD-directed treatment. The initial search was performed by XZ; key guideline statements, pivotal overlap syndrome (OVS) studies, and management-supporting references were checked against source records during manuscript preparation. No PRISMA flow, duplicate screening, or formal certainty grading was performed.

Evidence use is therefore classified throughout the manuscript as guideline-supported, direct OVS observational evidence, indirect COPD/OSA evidence, or physiologic inference. This classification is intended to prevent treatment-oriented statements from being read as guideline-level recommendations when they arise from adjacent COPD, OSA, or physiologic data rather than mechanism-stratified OVS trials.

## Diagnostic boundaries: confirmed COPD plus confirmed OSA or obstructive sleep apnea–hypopnea syndrome

OVS should be reserved for patients with both post-bronchodilator airflow obstruction compatible with COPD and objective evidence of OSA from polysomnography or an appropriate respiratory polygraphy/home sleep apnea test. Nocturnal desaturation in COPD without diagnostic OSA should be described as isolated nocturnal hypoxemia, not OVS. This distinction is not semantic: recent COPD data separated OSA-compatible OVS from isolated nocturnal hypoxemia, and earlier studies also showed that nocturnal desaturation can occur in COPD without dominant obstructive sleep apnea (Chaouat et al., 1995; Lewis et al., 2009; Lacasse et al., 2011; Marin et al., 2025).

COPD refers to post-bronchodilator airflow obstruction compatible with GOLD criteria, typically FEV_1_/FVC <0.70, interpreted with symptoms, exposure history, age, and local diagnostic standards (Global Initiative for Chronic Obstructive Lung Disease, 2026). Where local standards use lower-limit-of-normal criteria, spirometric confirmation should follow those standards. OSA refers to objective sleep-study evidence, conventionally AHI or respiratory event index (REI) ≥15 events/h, or ≥5 events/h with symptoms or relevant comorbidity (Kapur et al., 2017). Current AASM scoring criteria should be used for respiratory events and sleep-related hypoventilation (American Academy of Sleep Medicine, 2023).

Because OSA thresholds vary across guidelines and OVS cohorts, studies should report the AHI/REI threshold, hypopnea rule, symptom requirement, test platform, and whether OSAHS rather than OSA was used. In this review, OVS means spirometry-confirmed COPD plus objectively confirmed OSA by PSG or by RP/HSAT when guideline conditions for limited-channel testing are met. Oximetry alone should not diagnose OSA or OVS (Netzer et al., 2001; National Institute for Health and Care Excellence, 2021). The term COPD-OSAHS overlap syndrome is retained when NICE-specific recommendations are discussed (National Institute for Health and Care Excellence, 2021).

In OVS, diagnosis begins with objective confirmation of both component diseases, followed by mechanism-focused interpretation of the nocturnal tracing. Daytime assessment can include post-bronchodilator spirometry, resting SpO_2_, arterial or arterialized capillary blood gases when indicated, bicarbonate, BMI, exacerbation history, medication review, comorbid cardiopulmonary disease, and COPD treatment status (Global Initiative for Chronic Obstructive Lung Disease, 2026; Soler et al., 2017). Diffusing capacity, emphysema imaging, and echocardiography are useful when clinically indicated, especially when pulmonary hypertension or right-heart strain is suspected.

The AASM diagnostic testing guideline recommends PSG rather than HSAT in patients with significant cardiorespiratory disease, awake hypoventilation, or suspected sleep-related hypoventilation (Kapur et al., 2017). Limited-channel testing is most defensible in stable COPD with high pre-test probability of uncomplicated OSA, no hypercapnia, no suspected hypoventilation, and no unstable cardiopulmonary disease. PSG, RP/HSAT with CO_2_ monitoring, or specialist sleep evaluation is preferable when COPD is severe, PaCO_2_ or bicarbonate is elevated, oxygenation is disproportionate to AHI/REI, central events are suspected, an initial limited test is negative despite high suspicion, or titration is complex (Storre et al., 2007; Casey et al., 2007; Silva Júnior et al., 2017).

## Why AHI is insufficient in confirmed OVS

In objectively confirmed COPD-OSA overlap syndrome, the apnea-hypopnea index (AHI) often describes event frequency without capturing the physiological burden imposed by sleep on oxygenation and ventilation. Patients with modest AHI may spend prolonged periods at low oxygen saturation because COPD reduces oxygen reserve and impairs gas exchange, whereas patients with high AHI may show predominantly intermittent desaturation with relatively preserved between-event oxygenation. This mismatch between obstructive event counts, hypoxemic exposure, and ventilatory adequacy is the central problem addressed in this review (Weitzenblum and Chaouat, 2004; Malhotra et al., 2021).

AHI remains necessary for diagnosing and classifying OSA, but it does not capture baseline oxygenation, desaturation depth, event duration, delayed reoxygenation, sustained COPD-related desaturation, or CO_2_ retention. A moderate number of obstructive events can produce a large hypoxemic burden when oxygen reserve is poor; conversely, sustained gas-exchange impairment can produce high T90 even when AHI is modest.

AHI and REI differ operationally. PSG-based AHI uses sleep time, whereas many RP/HSAT indices use recording time and may underestimate event frequency when sleep efficiency is low. REM-related and supine-related event clustering, event duration, arousal burden, central apnea index, oxygen or positive airway pressure (PAP) status, and artifact handling are important to review when the numeric AHI does not fit the clinical picture. Hypopnea rule and sleep-time versus recording-time denominators can also change OSA diagnosis and OVS classification (American Academy of Sleep Medicine, 2023; Berry et al., 2012).

The available metrics answer different questions. ODI reflects how often oxygen saturation falls by a specified percentage, but it does not define the baseline from which those falls occur. T90/T88 and mean SpO_2_ describe cumulative and background oxygenation, whereas verified minimum SpO_2_ captures the deepest observed desaturation but is vulnerable to artifact. TcCO_2_, when available and clinically indicated, adds a direct window onto nocturnal ventilatory adequacy and sleep-related hypoventilation (American Academy of Sleep Medicine, 2023; Kapur et al., 2017).

### Mechanism 1: event-related upper-airway obstruction

Event-related upper-airway obstruction applies when recurrent obstructive apneas and hypopneas appear to drive most nocturnal hypoxemia. Typical clues are cyclical desaturation, high ODI, snoring or witnessed apneas, REM or supine clustering, and preserved between-event oxygenation; COPD may amplify each event by delaying reoxygenation.

Recurrent upper-airway obstruction adds intermittent hypoxemia, arousal-related sympathetic activation, intrathoracic pressure swings, and oxidative stress (Bradley and Floras, 2009; Javaheri et al., 2017). The dominant problem is repeated obstruction superimposed on reduced pulmonary reserve.

Mechanism-linked interpretation focuses on confirming obstruction and reassessing oxygenation and ventilation after treatment. CPAP is most relevant to OSA-predominant overlap without severe daytime hypercapnia or strong evidence of clinically important sleep-related hypoventilation (National Institute for Health and Care Excellence, 2021; Soler et al., 2017). NICE recommends CPAP as first-line treatment for COPD-OSAHS overlap when awake PaCO_2_ is 7.0 kPa or less (National Institute for Health and Care Excellence, 2021).

CPAP controls obstruction but may not correct sustained COPD-related hypoxemia. Modest expiratory pressure may offset intrinsic positive end-expiratory pressure and reduce work of breathing in selected COPD patients, although this evidence is mainly physiological and not OVS-specific (Petrof et al., 1990). Marked hyperinflation or emphysema may warrant individualized titration because excessive expiratory pressure can worsen mechanics or tolerance.

The OVS evidence base remains observational. Untreated OVS has been associated with higher mortality and COPD exacerbation-related hospitalization, whereas CPAP-treated OVS had outcomes closer to COPD without OSA (Marin et al., 2010). Real-world studies also associate PAP use or adherence with fewer severe exacerbations, hospitalizations, or deaths (Sterling et al., 2022; StanChina et al., 2013; Ioachimescu et al., 2020). Evidence basis: guideline-supported CPAP use plus direct OVS observational data, with adherence-related confounding, healthy-user bias, and selection bias.

### Mechanism 2: sustained low-baseline oxygenation and gas-exchange impairment

Sustained low-baseline oxygenation or gas-exchange impairment is suggested by low mean SpO_2_, plateau desaturation, high T90/T88, low resting SpO_2_ or DLCO, emphysema, pulmonary hypertension, or right-heart strain, indicating limited oxygen reserve rather than repeated obstructive dips alone.

COPD patients often enter sleep with reduced oxygen reserve and impaired ventilation–perfusion matching. On the steep portion of the oxyhemoglobin dissociation curve, small reductions in alveolar ventilation can produce large SpO_2_ falls (Weitzenblum and Chaouat, 2004; Flenley, 1985). Reduced chemosensitivity, functional residual capacity, and accessory muscle activity during sleep may sustain desaturation despite a modest AHI (Douglas, 1998; O'Donoghue et al., 2003).

Confirmed OSA remains present, but the pattern suggests that COPD-related gas-exchange impairment contributes substantially to nocturnal burden. Reassessment may focus on resting and nocturnal oxygenation, daytime gas exchange, COPD control, LTOT criteria, cardiopulmonary comorbidity, and CO_2_ status when hypoventilation is plausible.

Oxygen therapy is best framed by indication. LTOT for chronic severe resting hypoxemia is a standard COPD indication and need not be delayed because OVS is present (Global Initiative for Chronic Obstructive Lung Disease, 2026; Nocturnal Oxygen Therapy Trial Group, 1980; Medical Research Council Working Party, 1981; Jacobs et al., 2020). Local criteria commonly include PaO_2_ ≤55 mmHg or SpO_2_ ≤88%, or PaO_2_ 56–59 mmHg with pulmonary hypertension, edema, or polycythemia. Oxygen treats hypoxemia; OSA and hypoventilation require separate assessment.

Oxygen is not a substitute for treating the OSA component or suspected sleep-related hypoventilation because it neither splints the upper airway nor corrects ventilation. In sleep apnea with COPD, oxygen may improve saturation while leaving obstruction untreated and can prolong events or increase CO_2_ exposure in susceptible patients (Alford et al., 1986). COPD-related hypercapnia may also worsen through ventilation–perfusion redistribution, the Haldane effect, and reduced ventilatory drive (Robinson et al., 2000).

COPD oxygen trials support caution when severe resting hypoxemia is absent. LOTT found no benefit of long-term oxygen in stable COPD with moderate resting or exercise-induced desaturation, and INOX found no survival or LTOT-progression benefit from nocturnal oxygen in isolated nocturnal desaturation (Long-Term Oxygen Treatment Trial Research Group et al., 2016; Lacasse et al., 2020). These were not OVS trials but support mechanism-specific oxygen use and carbon dioxide safety monitoring. Evidence basis: guideline-supported LTOT, indirect COPD trials, and physiological inference.

### Mechanism 3: sleep-related hypoventilation and chronic hypercapnia

Sleep-related hypoventilation or chronic hypercapnia is suggested when nocturnal hypoxemia accompanies rising CO_2_, morning headache, elevated bicarbonate, cor pulmonale, recurrent acidotic exacerbations, or persistent daytime hypercapnia. It may be considered when T90/T88 is disproportionate to AHI/REI or mean SpO_2_ remains low despite apparently controlled obstruction.

AASM adult criteria define sleep hypoventilation as PaCO_2_, or a validated surrogate, >55 mmHg for ≥10 min, or a rise of ≥10 mmHg from awake supine values to >50 mmHg for ≥10 min (American Academy of Sleep Medicine, 2023). REM-related accessory muscle atonia increases reliance on a mechanically disadvantaged diaphragm and may promote hypoventilation and transient hypercapnia in COPD (Douglas, 1998; O'Donoghue et al., 2003).

CO_2_ monitoring may be useful when T90/T88 is disproportionate to AHI/REI, mean SpO_2_ remains low despite a low device-derived AHI, PaCO_2_ or bicarbonate is elevated, obesity-related ventilatory loading complicates attribution, COPD is severe, or residual hypoxemia persists on PAP. TcCO_2_ can identify REM-related or sustained hypoventilation; reports should specify the device, calibration, drift checks, artifact handling, and criteria. When TcCO_2_ is unavailable, awake PaCO_2_, bicarbonate, symptoms, and repeat testing may provide supporting context. End-tidal CO_2_ may be limited by leak, dead space, oxygen, and PAP/NIV conditions.

In patients with established COPD–OSA overlap, obesity and awake hypercapnia should not be labelled as obesity hypoventilation syndrome unless COPD and other causes are judged insufficient to explain the hypoventilation. In most cases, these findings are better described as obesity-related ventilatory loading superimposed on COPD–OSA, with attribution guided by COPD severity, arterial blood gases, and sleep-related carbon dioxide measurements (Mokhlesi et al., 2019).

Stable chronic hypercapnia, persistent hypercapnia after recovery from an acute exacerbation, recurrent acidotic exacerbations, or marked sleep-related hypoventilation may prompt ventilation-targeted evaluation rather than an assumption that CPAP alone is sufficient. NIV suitability warrants consideration within COPD and overlap-syndrome guideline contexts; treatment response may include PaCO_2_ or nocturnal TcCO_2_ control, oxygenation, sleep quality, symptoms, and exacerbation burden.

NICE recommends considering NIV rather than CPAP for COPD-OSAHS overlap when nocturnal hypoventilation accompanies severe awake hypercapnia, defined by the NICE-specific PaCO_2_ threshold of >7.0 kPa (National Institute for Health and Care Excellence, 2021). ATS and ERS guidance supports long-term home NIV for selected chronic hypercapnic COPD patients, with attention to CO_2_ reduction (Macrea et al., 2020; Ergan et al., 2019). Hypercapnic COPD trials provide indirect support, while severe OVS data remain observational and phenotype-dependent (Murphy et al., 2017; Köhnlein et al., 2014; Gao et al., 2024).

These data should be interpreted cautiously. Chronic hypercapnic COPD NIV trials are not OVS-specific randomized evidence, and a PaCO_2_ elevation during an acute exacerbation does not establish stable chronic hypercapnia. Evaluation for unrecognized OSA remains relevant before long-term NIV because expiratory pressure requirements and follow-up targets differ with overlap. When obstruction and hypoventilation coexist, expiratory pressure addresses obstruction, whereas pressure support, inspiratory pressure, backup-rate decisions, and synchrony address ventilation. Evidence basis: guideline-supported NIV contexts, indirect COPD trials, and limited direct OVS data.

## Post-treatment residual hypoxemia after PAP/NIV: a reassessment scenario

Residual hypoxemia after optimized PAP or NIV is not a baseline phenotype. It is a reassessment scenario that begins only after adequate nightly use, acceptable unintentional leak, control of residual obstructive events, appropriate expiratory and inspiratory pressures, assessment for central events, and evaluation of nocturnal CO_2_ when hypoventilation is plausible. A low device-derived AHI does not necessarily mean that OVS is controlled.

Persistent hypoxemia after apparently adequate CPAP or NIV may prompt reassessment of adherence, leak, residual events, REM/supine worsening, ventilatory support, CO_2_ retention, COPD control, cardiopulmonary comorbidity, LTOT criteria, oxygen fire risk, and carbon dioxide safety before oxygen is added.

A practical sequence is to verify oximetry signal quality and PAP adherence; review leak and device-reported residual events; distinguish residual obstructive events, central events, and REM/supine worsening; assess nocturnal hypoventilation using TcCO_2_ or daytime gas surrogates; reassess COPD control, pulmonary hypertension, heart failure, sedatives/opioids, and LTOT criteria; and only then consider adjunctive oxygen with carbon dioxide safety and fire-risk review. Device algorithms may misclassify events in complex respiratory disease, especially with leak or patient-ventilator asynchrony (Rabec et al., 2011; Schwab et al., 2013). Repeat oximetry, TcCO_2_ monitoring, RP/HSAT, or selected repeat PSG may be needed when symptoms, hypercapnia, or residual desaturation conflict with device indices.

Adjunctive nocturnal oxygen after optimized CPAP or NIV may be considered only when persistent nocturnal hypoxemia remains clinically significant according to local thresholds, symptoms, daytime gas exchange, pulmonary hypertension or right-heart strain, and carbon dioxide safety (National Institute for Health and Care Excellence, 2021). In patients with known or suspected CO_2_ retention, conservative titration and reassessment of blood gases may be appropriate after initiation or escalation. Home oxygen also requires safety assessment and education regarding smoking, open flames, ignition sources, tubing, and household fire risk (Jacobs et al., 2020).

## Mechanism-based phenotypes and treatable traits in confirmed OVS

The mechanisms above can be summarized as non-exclusive interpretive phenotypes or treatable traits after objective confirmation of COPD and OSA. They are not validated biological endotypes or treatment mandates, and prospective validation is required.

### OSA-predominant event-related phenotype

High AHI/REI and ODI, cyclical desaturation, REM/supine clustering, preserved between-event oxygenation, and near-normal awake PaCO_2_ suggest recurrent upper-airway obstruction as the dominant trait. The interpretation remains provisional because COPD can amplify event depth and delay reoxygenation even when obstruction is primary. Observational OVS studies associate PAP use or adherence with fewer hospitalizations, severe exacerbations, and health-care use, while CPAP-treated OVS had outcomes closer to COPD alone than untreated OVS (Marin et al., 2010; Sterling et al., 2022; StanChina et al., 2013; Ioachimescu et al., 2020).

### Gas-exchange-limited phenotype

Low mean SpO_2_, high T90/T88, plateau desaturation, low DLCO or resting SpO_2_, or pulmonary hypertension/right-heart strain despite modest AHI/REI suggest limited COPD-related reserve. The interpretive point is that cumulative hypoxemic exposure may be driven more by baseline gas-exchange impairment than by event frequency. Reassessment may include COPD control, daytime gas exchange, LTOT eligibility, cardiopulmonary comorbidity, and carbon dioxide safety. GOLD and ATS home-oxygen guidance provide the relevant COPD, LTOT, and safety context (Global Initiative for Chronic Obstructive Lung Disease, 2026; Jacobs et al., 2020).

### Hypoventilatory/hypercapnic phenotype

Elevated awake PaCO_2_ or bicarbonate, sustained or REM-related TcCO_2_ elevation, morning headache, cor pulmonale, or recurrent hypercapnic exacerbations suggest ventilatory insufficiency. A lower AHI after PAP does not establish correction of ventilation, and response assessment may also consider nocturnal carbon dioxide control, oxygenation, symptoms, sleep quality, and exacerbations. ATS guidance conditionally supports nocturnal NIV in selected chronic hypercapnic COPD patients and recommends OSA screening before NIV; this remains indirect COPD evidence rather than phenotype-stratified OVS trial evidence (Macrea et al., 2020).

### Residual hypoxemia after optimized PAP/NIV

**Figure 1:**
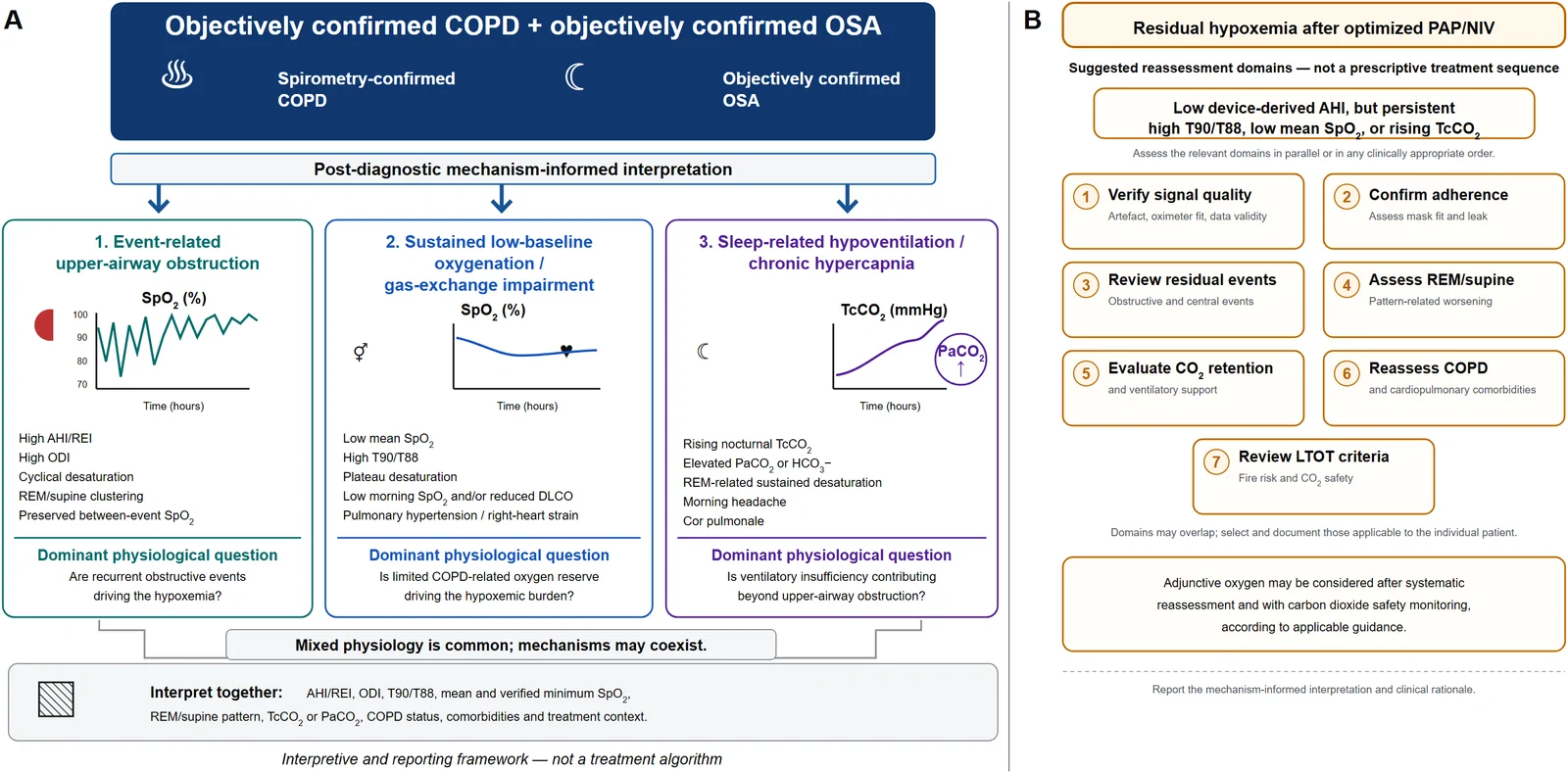
Mechanism-informed interpretation after chronic obstructive pulmonary disease and obstructive sleep apnea confirmation. **(A)** Diagnostic confirmation is separated from three baseline drivers in confirmed overlap syndrome. **(B)** Residual hypoxemia after PAP/NIV is treated as a post-treatment reassessment scenario rather than a baseline phenotype. Before adjunctive oxygen is considered, reassessment may include signal quality, adherence/leak, residual events, rapid eye movement/supine worsening, carbon dioxide status, COPD/comorbidity, long-term oxygen therapy criteria, oxygen fire risk, and carbon dioxide safety.

This is a post-treatment reassessment scenario, not a baseline phenotype. Persistent high T90/T88 or low mean SpO_2_ despite adequate use, acceptable leak, and controlled obstruction may reflect gas-exchange limitation, residual REM/supine obstruction, central events, inadequate ventilatory support, CO_2_ retention, COPD progression, pulmonary hypertension, heart failure, or medication effects. Device-derived event indices should be interpreted with oximetry quality and carbon dioxide data rather than in isolation. **Table 1** and **Figure 1** summarize reassessment before adjunctive oxygen is considered.

**Table 1:** Mechanism-oriented interpretive prompts and evidence hierarchy for nocturnal hypoxemia in confirmed overlap syndrome.

Interpretive scenario	Key clues to verify	Interpretive implication, reassessment focus, and evidence basis
Mechanism 1: event-related upper-airway obstruction	Cyclical desaturation, high ODI, REM/supine clustering, snoring or witnessed apneas, obesity, or resistant hypertension. Confirm obstruction with PSG or RP/HSAT; add TcCO_2_ when hypoventilation is plausible.	Obstruction-focused reassessment may include PAP adherence, leak, residual events, oxygenation, REM/supine pattern, and CO_2_ status when indicated. CPAP may not correct sustained low-baseline hypoxemia. Evidence: NICE NG202 and direct OVS observational PAP data (Marin et al., 2010; Sterling et al., 2022; StanChina et al., 2013; Ioachimescu et al., 2020). Limitation: non-randomized evidence with adherence and selection confounding.
Mechanism 2: sustained low-baseline oxygenation/gas-exchange impairment	Confirmed OSA with low baseline or mean SpO_2_, plateau desaturation, high T90/T88, emphysema, low FEV_1_/DLCO, pulmonary hypertension/right-heart strain, or low resting SpO_2_. Review awake gases, COPD control, LTOT criteria, and TcCO_2_ when needed.	Treat confirmed OSA while reassessing COPD control, gas exchange, LTOT eligibility, cardiopulmonary comorbidity, and CO_2_ status. Oxygen is not monotherapy for OSA or hypoventilation. Evidence: Global Initiative for Chronic Obstructive Lung Disease, 2026; Jacobs et al., 2020; NICE NG202; LOTT; INOX; and physiological inference. OVS-specific oxygen trials are limited.
Mechanism 3: sleep-related hypoventilation and chronic hypercapnia	Morning headache, cor pulmonale, recurrent acidotic exacerbations, persistent daytime hypercapnia, high bicarbonate, severe COPD, obesity-related ventilatory loading, and REM-related or sustained desaturation. Use awake gases and CO_2_-capable sleep testing when possible.	Ventilation-targeted assessment and NIV suitability warrant consideration rather than reliance on residual AHI alone. EPAP addresses obstruction; pressure support, inspiratory pressure, backup-rate decisions, and synchrony address ventilation. Response assessment may include PaCO_2_/TcCO_2_ control. Evidence: American Academy of Sleep Medicine, 2023; NICE NG202; ATS/ERS guidance; indirect COPD trials; and limited OVS data. No phenotype-stratified OVS randomized evidence is available.
Post-treatment residual hypoxemia after optimized PAP/NIV	Low device-derived AHI with persistent high T90/T88, low mean SpO_2_, or rising TcCO_2_ after adequate use, acceptable leak, and apparently controlled obstruction. Verify oximetry, device data, residual events, and comorbidity.	Not a baseline phenotype. Reassessment may include signal quality, adherence/leak, residual obstruction, central events, REM/supine worsening, ventilatory support, CO_2_ retention, COPD control, cardiopulmonary disease, medications, LTOT criteria, fire risk, and CO_2_ safety before oxygen is added. Evidence: NICE NG202; Jacobs et al., 2020; Rabec et al., 2011; Schwab et al., 2013; and physiological inference. Direct OVS thresholds are lacking.

Final management should follow local guidelines, patient-specific physiology, and reassessment of carbon dioxide safety. Evidence labels are interpretive and should not be read as guideline-level recommendations when based on indirect evidence or physiologic inference. AHI, apnea-hypopnea index; CPAP, continuous positive airway pressure; DLCO, diffusing capacity of the lung for carbon monoxide; EPAP, expiratory positive airway pressure; FEV_1_, forced expiratory volume in 1 s; LTOT, long-term oxygen therapy; NIV, noninvasive ventilation; ODI, oxygen desaturation index; OSA, obstructive sleep apnea; OVS, overlap syndrome; PAP, positive airway pressure; PSG, polysomnography; RP/HSAT, respiratory polygraphy/home sleep apnea testing; SpO_2_, peripheral oxygen saturation; TcCO_2_, transcutaneous carbon dioxide.

## Comorbidities and clinical modifiers that change interpretation and follow-up

Comorbidities and clinical modifiers should be recorded because they can change the physiological meaning of the same AHI/REI and determine the most appropriate follow-up target after PAP or NIV. A mechanism-oriented review should include BMI and body habitus, COPD phenotype and severity, resting SpO_2_, PaCO_2_ or bicarbonate, exacerbation timing, oxygen use, medication exposures, and cardiopulmonary comorbidity. NICE includes BMI, COPD exacerbation history, home oxygen therapy, comorbidities, oxygen saturation, blood gases, and current medicines among the information needed when assessing COPD-OSAHS overlap (National Institute for Health and Care Excellence, 2021). These variables do not replace PSG, RP/HSAT, or CO_2_-capable testing; rather, they determine whether nocturnal hypoxemia is interpreted as event-related obstruction, gas-exchange limitation, hypoventilation, or mixed physiology.

COPD phenotype and severity modify oxygen reserve. Emphysema, low DLCO, low resting SpO_2_, pulmonary hypertension or right-heart strain, chronic bronchitis, and frequent or recent exacerbations can shift a moderate AHI/REI toward high T90/T88, low mean SpO_2_, or plateau desaturation. Hyperinflation and increased end-expiratory lung volume may reduce upper-airway collapsibility through caudal traction in some emphysema-predominant patients (Biselli et al., 2015; Krachman et al., 2016), but this possible effect is inconsistent and should not be used to exclude OSA. Follow-up in this pattern should reassess COPD control, daytime gas exchange, LTOT eligibility, and pulmonary vascular or cardiac disease rather than escalating decisions on the basis of event counts alone.

Obesity and body habitus can increase upper-airway collapsibility and ventilatory load. Neck and abdominal obesity may promote REM- or supine-related obstruction, reduce respiratory-system compliance, and worsen sleep-related hypoventilation. In established COPD–OSA overlap, obesity, elevated bicarbonate, or awake hypercapnia are best described initially as obesity-related ventilatory loading. OHS may remain in the differential diagnosis only when COPD severity and other causes appear insufficient to explain hypoventilation, and attribution requires blood gases and sleep-related carbon dioxide assessment (Mokhlesi et al., 2019). CO_2_ assessment may be useful when high BMI, elevated bicarbonate, morning headache, cor pulmonale, or T90/T88 disproportionate to AHI/REI is present.

Cardiovascular comorbidity can reshape the nocturnal tracing. Heart failure, fluid overload, and rostral fluid shift may worsen obstruction, contribute to central events, or accentuate supine vulnerability (Redolfi et al., 2009; Yumino et al., 2010). Pulmonary hypertension, right-heart strain, resistant hypertension, arrhythmia, and ischemic heart disease may lower the threshold for careful oxygenation, CO_2_, and cardiopulmonary reassessment. A high central apnea index, Cheyne–Stokes breathing, unstable heart failure, or prominent central events warrants evaluation beyond a simple OVS framework (Javaheri et al., 2017; Randerath et al., 2018).

Recent COPD exacerbation, acute respiratory failure, and unstable cardiopulmonary disease require separate interpretation. PaCO_2_ elevation during an acute exacerbation should not be automatically classified as stable chronic hypercapnia, and residual nocturnal hypoxemia soon after an exacerbation may reflect transient airway inflammation, infection, fluid retention, deconditioning, or incomplete COPD optimization. Stable reassessment should document the timing of the last exacerbation, recent systemic corticosteroid or antibiotic use, changes in inhaled therapy, and whether ABG or TcCO_2_ findings persist after recovery (Global Initiative for Chronic Obstructive Lung Disease, 2026; Macrea et al., 2020).

Medication and substance exposures are part of mechanism assignment. Opioids, sedative-hypnotics, alcohol, anxiolytics, and other respiratory depressants may prolong events, blunt arousal or ventilatory responses, promote central events, and worsen hypoventilation. Neuromuscular weakness, severe restriction, and frailty can produce a similar ventilation-limited pattern. Persistent hypoxemia despite apparently adequate PAP/NIV, rising TcCO_2_, or discordance between symptoms and device-derived AHI warrants review of these factors (Casey et al., 2007; Randerath et al., 2018).

Foundational COPD care remains relevant because baseline mechanics and gas-exchange reserve shape nocturnal vulnerability. Smoking cessation, inhaled pharmacotherapy tailored to symptoms and exacerbation risk, vaccination, pulmonary rehabilitation, and comorbidity management can improve the substrate on which OSA and hypoventilation are superimposed (Global Initiative for Chronic Obstructive Lung Disease, 2026; Spruit et al., 2013). Bronchodilation may reduce hyperinflation, improve diaphragmatic mechanics, and lessen work of breathing during sleep, although direct evidence on OVS sleep-breathing outcomes remains limited (Rossi et al., 2015).

Oxygen-related modifiers should be documented separately. Supplemental oxygen during diagnostic testing or PAP/NIV follow-up can change desaturation-based hypopnea scoring, T90/T88, mean SpO_2_, and nadir SpO_2_ without correcting obstruction or hypoventilation. Active smoking, open flames, household ignition sources, tubing risks, and patient education alter oxygen safety and follow-up planning (Jacobs et al., 2020). In patients with known or suspected CO_2_ retention, oxygen reassessment should include carbon dioxide safety rather than SpO_2_ targets alone.

Taken together, modifier-informed follow-up should match the dominant physiological question. OSA-predominant patterns prioritize adherence, leak, residual events, REM/supine clustering, and post-PAP oxygenation. Gas-exchange-limited patterns prioritize resting and nocturnal oxygenation, ABG or capillary gases, LTOT criteria, pulmonary hypertension, and heart failure. Hypoventilatory or hypercapnic patterns prioritize PaCO_2_, bicarbonate, TcCO_2_, NIV settings, synchrony, and backup-rate considerations. Mixed patterns require parallel reassessment rather than assuming that a low device-derived AHI represents controlled OVS. Evidence basis: guideline-supported assessment domains, direct and indirect physiologic data, and limited OVS-specific validation.

## Candidate biomarkers and translational markers: useful signals but not diagnostic substitutes

Serum bicarbonate, PaCO_2_, and TcCO_2_ are the most practical ventilation-related signals. They do not diagnose OVS, but flag possible hypoventilation or chronic hypercapnia when desaturation is disproportionate to AHI/REI. The ATS OHS guideline uses serum HCO_3_^−^ ≥27 mmol/L to prompt ABG confirmation in obese adults with sleep-disordered breathing (Mokhlesi et al., 2019). In COPD, attribution must be cautious because airflow obstruction, recent exacerbation, oxygen exposure, diuretics, obesity-related ventilatory loading and, in selected differential evaluations, OHS can influence bicarbonate or PaCO_2_; TcCO_2_ adds nocturnal physiologic context.

Inflammatory biomarkers are promising but not mature diagnostic tools. Sanchez-Azofra et al. reported higher IL-6, hs-CRP, and G-CSF in OVS and COPD than in controls or OSA alone, and higher leukocytes and neutrophils in OVS than in COPD, OSA, or controls. They proposed IL-6, G-CSF, and hs-CRP as possible COPD-screening aids among OSA patients (Sanchez-Azofra et al., 2023). This remains a candidate signal, not an OVS-specific biomarker, and may be confounded by smoking, obesity, infection, exacerbation, corticosteroids, or cardiovascular disease.

Blood eosinophils are better framed as a COPD treatable trait. COPD guidance uses eosinophil counts to estimate benefit from inhaled corticosteroid-containing regimens and reflect an eosinophilic inflammatory phenotype in exacerbation-prone patients (Global Initiative for Chronic Obstructive Lung Disease, 2026). In confirmed OVS, eosinophils may personalize COPD therapy, but should not diagnose OVS or distinguish it from OSA or COPD alone.

Hypoxic burden, T90/T88, and total nocturnal hypoxemic burden are physiological biomarkers or risk markers. Event-associated hypoxic burden integrates desaturation depth and duration, whereas T90/T88 and total nocturnal burden capture sustained low-baseline oxygenation from COPD gas-exchange limitation (Azarbarzin et al., 2019; Malhotra et al., 2021). Kendzerska et al. showed that COPD combined with nocturnal hypoxemia was associated with higher hazards of cardiovascular events and all-cause mortality, supporting oxygenation-burden reporting beyond AHI alone (Kendzerska et al., 2019). These markers should complement, not replace, objective confirmation of COPD and OSA.

## Core versus extended reporting items

**Table 2** summarizes the proposed core and extended reporting items and their evidence basis. For routine reporting, the core minimum should include the AHI or REI method, hypopnea rule, ODI threshold, T90 or T88 with denominator, mean and verified minimum SpO_2_, oxygen/PAP/NIV status, oximetry averaging time or artifact handling, and REM/supine time when available. Extended reporting is appropriate for research and complex OVS assessment, especially hypoxic burden, event duration, TcCO_2_/ETCO_2_, device data, and cardiopulmonary modifiers.

**Table 2:** Core versus extended oxygenation and ventilation reporting items and evidence basis for research and complex clinical assessment in COPD-OSA overlap syndrome.

Reporting item	What it adds beyond AHI/REI	Minimum reporting and COPD-specific caveat	Evidence basis/key sources
AHI/REI (core)	Confirms OSA and quantifies obstructive event frequency.	Report PSG sleep-time AHI versus RP/HSAT recording-time REI, hypopnea rule, symptom threshold, platform, oxygen/PAP status, REM/supine time, and central apnea index. In COPD, a modest AHI/REI may coexist with substantial hypoxemic exposure.	(Kapur et al., 2017; American Academy of Sleep Medicine, 2023; NICE NG202; Global Initiative for Chronic Obstructive Lung Disease, 2026).
ODI (core)	Describes intermittent oxygen desaturation frequency and helps identify event-linked hypoxemia.	Specify 3% or 4% threshold, denominator, valid signal time, artifact handling, and oxygen/PAP status. ODI cannot replace AHI/REI or CO_2_ assessment and may miss sustained COPD-related hypoxemia.	(American Academy of Sleep Medicine, 2023; Berry et al., 2012; physiologic inference.
T90/T88 (core)	Quantifies cumulative time below a saturation threshold and captures sustained low-baseline oxygenation.	Report threshold, denominator, valid oximetry time, artifact handling, averaging time, and oxygen/PAP status. Interpret with baseline SpO_2_, resting oxygenation, altitude, LTOT criteria, and CO_2_ status.	(Kendzerska et al., 2019; Bae et al., 2021; Global Initiative for Chronic Obstructive Lung Disease, 2026; NICE NG202; Jacobs et al., 2020).
Mean and verified minimum SpO_2_ (core)	Summarizes background oxygenation and the deepest observed desaturation.	Report mean SpO_2_ and manually verified nadir SpO_2_. Mean SpO_2_ may mask brief deep dips; nadir SpO_2_ is artifact-sensitive and should not drive decisions unless signal quality is confirmed.	(American Academy of Sleep Medicine, 2023; Weitzenblum and Chaouat, 2004; O'Donoghue et al., 2003).
REM/supine time and clustering (core when available)	Identifies sleep-stage and positional vulnerability that may explain discordance between AHI/REI and hypoxemia.	Report REM time, supine time, REM AHI and supine AHI when available, and whether desaturation is clustered or sustained. Limited REM/supine sleep can underestimate severity.	(American Academy of Sleep Medicine, 2023; Douglas, 1998; O'Donoghue et al., 2003).
Oxygen/PAP/NIV status and oximetry settings (core)	Prevents misinterpretation of desaturation-based scoring, T90/T88, mean SpO_2_, and nadir SpO_2_.	Record oxygen flow, delivery route, bleed-in location, PAP/NIV mode and settings when applicable, oximeter averaging time, sampling/artifact rules, and valid recording time. Oxygen can improve SpO_2_ without correcting obstruction or hypoventilation.	NICE NG202; Jacobs et al., 2020; American Academy of Sleep Medicine, 2023; Alford et al., 1986; Robinson et al., 2000.
TcCO_2_/ETCO_2_ (additional when indicated)	Assesses ventilatory adequacy and detects sleep-related hypoventilation or persistent hypercapnia.	Use when hypoxemia is disproportionate to AHI/REI, hypercapnia is present, obesity-related ventilatory loading complicates attribution, residual hypoxemia persists on PAP, or NIV is initiated/adjusted. Report device, calibration, drift checks, artifact handling, and hypoventilation criteria.	(American Academy of Sleep Medicine, 2023; Mokhlesi et al., 2019; NICE NG202; Macrea et al., 2020; Ergan et al., 2019).
Hypoxic burden (research/additional)	Integrates desaturation depth and duration and may better reflect physiologic stress than event frequency alone.	Specify event-associated versus total threshold-based burden, calculation method, denominator, and oxygen/PAP status. Promising for risk stratification, but not a validated OVS treatment target.	(Azarbarzin et al., 2019; Malhotra et al., 2021; Kendzerska et al., 2019; Bae et al., 2021).
Event duration/reoxygenation pattern (research/additional)	Highlights prolonged obstruction or delayed recovery that can enlarge hypoxemic exposure despite similar AHI/REI.	Report event duration distribution and recovery pattern when available. Methods are not standardized for routine OVS management and should be interpreted with baseline SpO_2_ and gas-exchange reserve.	(Malhotra et al., 2021; Weitzenblum and Chaouat, 2004; O'Donoghue et al., 2003).

T90 and T88 denote time or percentage of recording/sleep time with SpO_2_ <90% or <88%, respectively. ODI should state whether a 3% or 4% desaturation threshold was used. Hypoxic burden should specify whether it is event-associated or threshold-based total nocturnal hypoxemic burden. Evidence-basis terms follow the Methods section and are used to distinguish guideline-supported statements, direct OVS observational evidence, indirect COPD/OSA evidence, and physiologic inference. AASM, American Academy of Sleep Medicine; AHI, apnea-hypopnea index; COPD, chronic obstructive pulmonary disease; ETCO_2_, end-tidal carbon dioxide; LTOT, long-term oxygen therapy; NIV, noninvasive ventilation; ODI, oxygen desaturation index; OSA, obstructive sleep apnea; OVS, overlap syndrome; PAP, positive airway pressure; PSG, polysomnography; REI, respiratory event index; REM, rapid eye movement; RP/HSAT, respiratory polygraphy/home sleep apnea testing; SpO_2_, peripheral oxygen saturation; TcCO_2_, transcutaneous carbon dioxide.

No single nocturnal SpO_2_ threshold has been validated as a universal treatment trigger in OVS. Reports should state the threshold used, such as T90 or T88, and interpret it with mean SpO_2_, nadir SpO_2_, resting oxygenation, altitude, oxygen/PAP status, CO_2_ retention, LTOT criteria, symptoms, and cardiopulmonary comorbidity. T90/T88 reporting should state whether the denominator is total sleep time, recording time, or valid oximetry time.

Supplemental oxygen during diagnostic testing requires documentation because it may reduce desaturation-based hypopnea scoring and alter T90/T88, mean SpO_2_, and nadir SpO_2_ without correcting obstruction or hypoventilation. Oxygen flow rate, delivery route, and bleed-in location during PAP/NIV should be recorded when applicable.

## Worked interpretive examples

After COPD and OSA are confirmed, the central clinical task is to identify the most plausible driver or drivers of nocturnal hypoxemia. The following examples are non-exclusive interpretive scenarios; they may coexist and may change after treatment. **Table 1** summarizes mechanism-oriented prompts and reassessment priorities.

Example 1: event-related obstruction. Data pattern: Confirmed COPD and OSA with AHI 42 events/h, high ODI, cyclic desaturation, preserved between-event SpO_2_, and normal PaCO_2_. Interpretation: Recurrent upper-airway obstruction probably explains most nocturnal hypoxemia. Reassessment may include PAP response, leak, residual events, mean SpO_2_, T90/T88, REM/supine clustering, symptoms, and CO_2_ status when indicated. Evidence caveat: guideline-based OSA/overlap principles and observational OVS PAP data, not OVS randomized trials.

Example 2: gas-exchange-limited OVS. Data pattern: Confirmed OVS with AHI 12 events/h, mean SpO_2_ 88%, T90 60%, plateau desaturation, low DLCO, and low resting SpO_2_. Interpretation: Limited gas-exchange reserve probably contributes more than obstruction alone; OSA treatment may reduce dips without normalizing baseline oxygenation. Reassessment warrants review of COPD control, daytime gases, LTOT criteria, cardiopulmonary disease, and CO_2_ status. Evidence caveat: COPD/LTOT guidance, indirect oxygen evidence, and physiological inference; OVS-specific oxygen trials are limited.

Example 3: hypoventilatory or hypercapnic OVS. Data pattern: Confirmed OVS with AHI 16 events/h, prolonged REM-related desaturation, morning headache, elevated bicarbonate, awake PaCO_2_ 52 mmHg, and TcCO_2_ >55 mmHg for at least 10 min during REM sleep. Interpretation: Sleep-related hypoventilation or chronic hypercapnia accompanies obstruction; a lower residual AHI would not establish corrected ventilation. Reassessment may include gases, bicarbonate, TcCO_2_ trend, COPD stability, obesity-related ventilatory loading, sedatives/opioids, NIV suitability, and CO_2_ safety. OHS warrants differential evaluation only if COPD and other causes appear insufficient. Evidence caveat: AASM criteria, guideline contexts, indirect COPD trials, and limited OVS data; phenotype-stratified randomized evidence is lacking.

Example 4: residual hypoxemia after PAP. Data pattern: After apparently adequate CPAP/NIV use, device-derived AHI is <5 events/h, but T90 remains high and TcCO_2_ rises during REM sleep. Interpretation: This is not simply a residual oxygen need; possible explanations include leak, residual REM/supine obstruction, central events, inadequate ventilatory support, gas-exchange limitation, CO_2_ retention, and cardiopulmonary disease. Reassessment warrants review of device data, ventilation, COPD and cardiac status, medications, LTOT criteria, fire risk, and CO_2_ safety before oxygen is added. Evidence caveat: guideline follow-up principles, device limitations, oxygen-safety guidance, and physiological inference; direct OVS thresholds are lacking.

## Clinical outcome evidence: what is known and what remains indirect

### PAP/CPAP outcomes in OVS

The clinical outcome evidence for PAP/CPAP in OVS comes mainly from observational cohorts and real-world studies, rather than adequately powered OVS-specific randomized controlled trials. In the landmark cohort by Marin et al., untreated OVS was associated with increased all-cause mortality and COPD exacerbation-related hospitalization compared with COPD alone, whereas CPAP-treated OVS showed lower mortality and fewer COPD-related hospitalizations, with outcomes closer to COPD without OSA (Marin et al., 2010). In a large real-world analysis, PAP-adherent patients with concomitant OSA and COPD had lower rates of emergency room visits, hospitalizations, severe acute exacerbations, and total health-care costs than non-adherent patients (Sterling et al., 2022). Evidence level: direct OVS observational/real-world evidence, with plausible confounding by adherence, selection, and healthy-user effects; not OVS-specific randomized outcome evidence.

### NIV outcomes in hypercapnic or severe OVS

OVS-specific NIV outcome evidence remains limited. Gao et al. studied severe OVS and reported that NIV was associated with improved survival and reduced rehospitalization, but the study was non-randomized and the authors emphasized the need for confirmatory studies (Gao et al., 2024). Evidence from long-term NIV trials in chronic hypercapnic COPD, including PaCO_2_-targeted NIV in stable severe COPD and home NIV after acute hypercapnic exacerbation, provides indirect physiologic and clinical support for ventilation-targeted therapy when OVS includes persistent hypercapnia or sleep-related hypoventilation; however, chronic hypercapnic COPD data should not be treated as OVS-specific evidence (Köhnlein et al., 2014; Murphy et al., 2017; Macrea et al., 2020; Ergan et al., 2019). Evidence level: limited direct OVS observational evidence plus indirect randomized COPD evidence and guideline-supported chronic hypercapnic COPD recommendations.

### Oxygen outcomes and safety

Oxygen evidence should be separated by indication. First, LTOT is supported for COPD with chronic severe resting hypoxemia, and ATS home oxygen guidance defines severe chronic resting hypoxemia as PaO_2_ ≤55 mmHg or SpO_2_ ≤88%, or PaO_2_ 56–59 mmHg/SpO_2_ 89% plus edema, Hct ≥55%, or P pulmonale; this is a COPD guideline-supported indication, not an OVS-specific oxygen trial result (Nocturnal Oxygen Therapy Trial Group, 1980; Medical Research Council Working Party, 1981; Jacobs et al., 2020; Global Initiative for Chronic Obstructive Lung Disease, 2026). Second, oxygen should not be used as monotherapy for the OSA component. In OSA, CPAP compared with nocturnal oxygen significantly lowered blood pressure, whereas nocturnal oxygen did not, and systematic review evidence shows that oxygen can improve saturation but is less effective than CPAP in lowering AHI and may prolong obstructive events (Gottlieb et al., 2014; Mehta et al., 2013). Third, oxygen in OVS/COPD requires CO_2_ safety. Alford et al. provide direct early evidence in COPD plus sleep apnea that acute oxygen can improve saturation while increasing obstructive event duration and CO_2_ exposure; in COPD, oxygen-induced hypercapnia is linked to ventilation-perfusion redistribution, the Haldane effect, and changes in ventilatory drive (Alford et al., 1986; Robinson et al., 2000). LOTT showed no benefit of long-term oxygen on death or first hospitalization in stable COPD with moderate desaturation, and INOX showed no significant benefit of nocturnal oxygen on survival or progression to LTOT in COPD with nocturnal desaturation not qualifying for LTOT, although INOX was underpowered (Long-Term Oxygen Treatment Trial Research Group et al., 2016; Lacasse et al., 2020). Evidence level: guideline-supported LTOT for severe chronic resting COPD hypoxemia; indirect OSA and COPD randomized evidence against oxygen as OSA monotherapy or routine use in moderate/nocturnal-only desaturation; direct physiologic early OVS/COPD-sleep apnea safety evidence and COPD physiologic evidence for CO_2_ monitoring.

## Discussion

What this framework adds. This Review does not restate the definition, epidemiology, or standard treatment of COPD-OSA overlap syndrome, which are covered in previous reviews, systematic reviews, and research statements (McNicholas, 2009; Owens and Malhotra, 2010; McNicholas et al., 2013; Budhiraja et al., 2015; Shawon et al., 2017; McNicholas, 2017; Malhotra et al., 2018; Wei et al., 2026; Voulgaris et al., 2026). Its contribution is to organize AHI–oxygenation–ventilation discordance after objective confirmation into three testable baseline mechanisms and a post-treatment reassessment scenario. Event frequency, cumulative hypoxemic exposure, and ventilatory adequacy answer related but distinct questions, so a single severity number may conceal clinically important heterogeneity. Rather than replacing AHI, the framework links discordant findings to hypotheses that can be checked against oximetry quality, sleep stage and position, daytime gases, CO_2_ monitoring, device data, COPD status, and comorbid disease. Mixed physiology is expected, and the dominant mechanism may change after treatment. The framework is therefore interpretive and falsifiable, not algorithmic.

### Where evidence is strong

The strongest elements are diagnostic boundaries, testing choices, and minimum reporting requirements. COPD confirmation rests on post-bronchodilator spirometry, while OSA requires objective sleep-study evidence. AASM guidance supports PSG rather than HSAT in significant cardiorespiratory disease, awake hypoventilation, or suspected sleep-related hypoventilation and provides operational scoring criteria for respiratory events and sleep hypoventilation. NICE gives explicit COPD-OSAHS overlap guidance, including CPAP when awake PaCO_2_ is 7.0 kPa or less and consideration of NIV when nocturnal hypoventilation accompanies severe awake hypercapnia. ATS/ERS NIV, ATS home-oxygen, and ATS OHS guidance support chronic hypercapnia assessment, oxygen indications, and cautious differential attribution of obesity-related hypoventilation. These sources do not validate the proposed phenotypes or define universal nocturnal oxygenation thresholds, but they provide a robust foundation for selecting PSG, carbon dioxide-capable testing, blood gases, oxygen assessment, or NIV evaluation in higher-risk patients (Global Initiative for Chronic Obstructive Lung Disease, 2026; Kapur et al., 2017; American Academy of Sleep Medicine, 2023; National Institute for Health and Care Excellence, 2021; Mokhlesi et al., 2019; Macrea et al., 2020; Ergan et al., 2019; Jacobs et al., 2020).

### Where evidence is moderate or observational

PAP/CPAP outcome evidence in OVS is clinically important but mainly non-randomized. Cohort and real-world studies associate treatment or adherence with lower mortality, fewer COPD-related hospitalizations or exacerbations, and lower health-care use (Marin et al., 2010; StanChina et al., 2013; Ioachimescu et al., 2020; Sterling et al., 2022). These associations should not be read as proof that all OVS mechanisms respond equally to CPAP because adherence, healthy-user, selection, COPD-severity, comorbidity, smoking, oxygen use, access, and tolerance may confound benefit. The data support treating the OSA component but cannot determine whether residual T90/T88, hypoxic burden, or TcCO_2_ trajectories warrant CPAP, NIV, oxygen, or COPD-treatment intensification in a mechanism-stratified way.

### Where evidence is indirect

NIV and oxygen implications are especially prone to extrapolation. NIV support comes largely from chronic hypercapnic COPD trials and guidelines rather than OVS randomized trials stratified by AHI–oxygenation–ventilation pattern or TcCO_2_-defined mechanism. These data are relevant to persistent hypercapnia or sleep-related hypoventilation but do not prove that TcCO_2_-guided NIV escalation is superior to AHI-guided PAP escalation (Köhnlein et al., 2014; Murphy et al., 2017; Macrea et al., 2020; Ergan et al., 2019). Oxygen evidence is also indirect: COPD trials and guidance address chronic resting hypoxemia, while OSA studies show improved saturation without correction of obstruction and possible inferiority to CPAP for key outcomes (Nocturnal Oxygen Therapy Trial Group, 1980; Medical Research Council Working Party, 1981; Gottlieb et al., 2014; Mehta et al., 2013; Jacobs et al., 2020). LOTT and INOX caution against routine oxygen in moderate or nocturnal-only COPD desaturation, and early COPD-sleep-apnea physiology indicates that oxygen may increase carbon dioxide exposure in susceptible patients (Long-Term Oxygen Treatment Trial Research Group et al., 2016; Lacasse et al., 2020; Alford et al., 1986; Robinson et al., 2000). These findings support cautious, indication-specific interpretation rather than direct transfer of COPD or OSA treatment thresholds to OVS. Treatment thresholds for hypoxic burden and residual T90/T88 remain unproven.

### Future research

The framework requires prospective testing in well-characterized OVS cohorts. Priorities include whether residual T90/T88 after optimized PAP predicts mortality, exacerbations, pulmonary hypertension, or hospitalization independently of AHI, lung function, and comorbidity; whether event-associated and total nocturnal hypoxemic burden distinguish obstruction, gas-exchange limitation, and hypoventilation; and whether TcCO_2_-guided ventilation strategies improve outcomes. Candidate physiological and translational markers, including bicarbonate, PaCO_2_, TcCO_2_ trajectories, diffusing capacity, imaging, inflammatory markers, and hypoxic-burden signatures, also require validation before they can define phenotypes or treatment targets. Mechanism-stratified trials should compare obstruction-focused PAP, ventilation-targeted NIV, adjunctive oxygen with carbon dioxide safety, and intensified COPD care. Standardized reporting of AHI/REI method, hypopnea rule, T90/T88 denominator, mean and minimum SpO_2_, ODI threshold, REM/supine exposure, treatment context, and carbon dioxide measurements is essential for evaluating these mechanisms and their prognostic value.

## Limitations of this review and framework

This review has several limitations. The literature search was restricted to one bibliographic database and English-language adult studies, and study selection was purposive rather than systematic. Duplicate screening, formal risk-of-bias assessment, certainty grading, and quantitative synthesis were not performed. The underlying OVS literature is heterogeneous in diagnostic definitions, hypopnea rules, oximetry thresholds, carbon dioxide monitoring, and treatment exposure. Most PAP outcome data are observational, whereas much of the NIV and oxygen evidence is extrapolated from COPD or OSA populations. The proposed mechanistic categories and reporting items therefore require prospective assessment before routine adoption.

## Conclusion

AHI remains important for diagnosing OSA, but nocturnal hypoxemia and ventilatory inadequacy in COPD-OSA overlap syndrome should be interpreted as markers of disease interaction rather than simple extensions of AHI. T90/T88, mean nocturnal SpO_2_, verified minimum SpO_2_, ODI, hypoxic burden, event duration, and TcCO_2_ may help organize a mechanism-informed interpretation when considered with lung function, daytime gas exchange, symptoms, comorbidities, and treatment context. The framework therefore emphasizes structured interpretation and transparent reporting of event-related obstruction, sustained low-baseline oxygenation or gas-exchange impairment, sleep-related hypoventilation/chronic hypercapnia, and post-treatment residual hypoxemia as reassessment signals. At minimum, reports should specify the AHI or respiratory event index method, hypopnea rule, oxygen desaturation index threshold, T90/T88 denominator, mean and verified minimum oxygen saturation, rapid eye movement and supine exposure, and treatment context; transcutaneous carbon dioxide, hypoxic burden, and event-duration measures should be considered extended or research-oriented items.

However, these categories should not be interpreted as validated biological endotypes or as instructions for selecting CPAP, NIV, oxygen, or COPD-directed treatment. The available evidence is strongest for diagnostic boundaries and reporting principles, but mechanism-stratified outcome data in OVS remain limited. This framework should be regarded as a structured interpretive and reporting approach rather than a treatment algorithm. Its clinical utility, reproducibility, and prognostic value require prospective validation in well-characterized OVS cohorts.

**Supplementary Material**: **Supplementary Material 1** contains the PubMed search syntax, guideline and hand-searched sources, narrative selection priorities, evidence-use statement, and SANRA-based checklist.

## Supplementary material

Supplementary Material 1 Search strategy, literature selection details, and SANRA-based checklist.

## References

[B1] Alford N. J. Fletcher E. C. Nickeson D. (1986). Acute oxygen in patients with sleep apnea and COPD. Chest 89, 30–38. doi: 10.1378/chest.89.1.30 3079693

[B2] American Academy of Sleep Medicine (2023). The AASM Manual for the Scoring of Sleep and Associated Events: Rules, Terminology and Technical Specifications, Version 3 (Darien, IL: American Academy of Sleep Medicine). Available online at: https://shop.aasm.org/products/aasm-scoring-manual-3-ebook (Accessed August 3, 2026).

[B3] Azarbarzin A. Sands S. A. Stone K. L. Taranto-Montemurro L. Messineo L. Terrill P. I. . (2019). The hypoxic burden of sleep apnoea predicts cardiovascular disease-related mortality: the Osteoporotic Fractures in Men Study and the Sleep Heart Health Study. Eur. Heart J. 40, 1149–1157. doi: 10.1093/eurheartj/ehy624 30376054 PMC6451769

[B4] Bae E. Kwak N. Choi S. M. Lee J. Park Y. S. Lee C. H. . (2021). Mortality prediction in chronic obstructive pulmonary disease and obstructive sleep apnea. Sleep Med. 87, 143–150. doi: 10.1016/j.sleep.2021.09.011 34607112

[B5] Baethge C. Goldbeck-Wood S. Mertens S. (2019). SANRA-a scale for the quality assessment of narrative review articles. Res. Integr. Peer Rev. 4, 5. doi: 10.1186/s41073-019-0064-8 30962953 PMC6434870

[B6] Berry R. B. Budhiraja R. Gottlieb D. J. Gozal D. Iber C. Kapur V. K. . (2012). Rules for scoring respiratory events in sleep: update of the 2007 AASM Manual for the Scoring of Sleep and Associated Events. Deliberations of the Sleep Apnea Definitions Task Force of the American Academy of Sleep Medicine. J. Clin. Sleep Med. 8, 597–619. doi: 10.5664/jcsm.2172 23066376 PMC3459210

[B7] Biselli P. Grossman P. R. Kirkness J. P. Patil S. P. Smith P. L. Schwartz A. R. . (2015). The effect of increased lung volume in chronic obstructive pulmonary disease on upper airway obstruction during sleep. J. Appl. Physiol. (1985) 119, 266–271. doi: 10.1152/japplphysiol.00455.2014 26048975 PMC4526705

[B8] Bradley T. D. Floras J. S. (2009). Obstructive sleep apnoea and its cardiovascular consequences. Lancet 373, 82–93. doi: 10.1016/S0140-6736(08)61622-0 19101028

[B9] Budhiraja R. Siddiqi T. A. Quan S. F. (2015). Sleep disorders in chronic obstructive pulmonary disease: etiology, impact, and management. J. Clin. Sleep Med. 11, 259–270. doi: 10.5664/jcsm.4540 25700872 PMC4346647

[B10] Casey K. R. Cantillo K. O. Brown L. K. (2007). Sleep-related hypoventilation/hypoxemic syndromes. Chest 131, 1936–1948. doi: 10.1378/chest.06-2334 17565028

[B11] Chaouat A. Weitzenblum E. Krieger J. Ifoundza T. Oswald M. Kessler R. (1995). Association of chronic obstructive pulmonary disease and sleep apnea syndrome. Am. J. Respir. Crit. Care Med. 151, 82–86. doi: 10.1164/ajrccm.151.1.7812577 7812577

[B12] Douglas N. J. (1998). Sleep in patients with chronic obstructive pulmonary disease. Clin. Chest Med. 19, 115–125. doi: 10.1016/s0272-5231(05)70436-6 9554222

[B13] Ergan B. Oczkowski S. Rochwerg B. Carlucci A. Chatwin M. Clini E. . (2019). European Respiratory Society guidelines on long-term home non-invasive ventilation for management of COPD. Eur. Respir. J. 54, 1901003. doi: 10.1183/13993003.01003-2019 31467119

[B14] Flenley D. C. (1985). Sleep in chronic obstructive lung disease. Clin. Chest Med. 6, 651–661. doi: 10.1016/s0272-5231(21)00402-0 2935359

[B15] Gao Y. Fan Z. Zhang H. Jiao Y. Covassin N. Li F. . (2024). Prognostic efficacy of non-invasive ventilation in patients with overlap syndrome: chronic obstructive pulmonary disease and obstructive sleep apnea. J. Thorac. Dis. 16, 4947–4956. doi: 10.21037/jtd-24-390 39268122 PMC11388228

[B16] Global Initiative for Chronic Obstructive Lung Disease (2026). Global Strategy for Prevention, Diagnosis and Management of COPD: 2026 Report. Available online at: https://goldcopd.org/2026-gold-report-and-pocket-guide/.

[B17] Gottlieb D. J. Punjabi N. M. Mehra R. Patel S. R. Quan S. F. Babineau D. C. . (2014). CPAP versus oxygen in obstructive sleep apnea. N. Engl. J. Med. 370, 2276–2285. doi: 10.1056/NEJMoa1306766 24918372 PMC4172401

[B18] Ioachimescu O. C. Janocko N. J. Ciavatta M. M. Howard M. Warnock M. V. (2020). Obstructive Lung Disease and Obstructive Sleep Apnea (OLDOSA) cohort study: 10-year assessment. J. Clin. Sleep Med. 16, 267–277. doi: 10.5664/jcsm.8180 31992433 PMC7053033

[B19] Jacobs S. S. Krishnan J. A. Lederer D. J. Ghazipura M. Hossain T. Tan A. M. . (2020). Home oxygen therapy for adults with chronic lung disease. An official american thoracic society clinical practice guideline. Am. J. Respir. Crit. Care Med. 202, e121–e141. doi: 10.1164/rccm.202009-3608ST 33185464 PMC7667898

[B20] Javaheri S. Barbe F. Campos-Rodriguez F. Dempsey J. A. Khayat R. Javaheri S. . (2017). Sleep apnea: types, mechanisms, and clinical cardiovascular consequences. J. Am. Coll. Cardiol. 69, 841–848. doi: 10.1016/j.jacc.2016.11.069 28209226 PMC5393905

[B21] Kapur V. K. Auckley D. H. Chowdhuri S. Kuhlmann D. C. Mehra R. Ramar K. . (2017). Clinical practice guideline for diagnostic testing for adult obstructive sleep apnea: an american academy of sleep medicine clinical practice guideline. J. Clin. Sleep Med. 13, 479–504. doi: 10.5664/jcsm.6506 28162150 PMC5337595

[B22] Kendzerska T. Leung R. S. Aaron S. D. Ayas N. Sandoz J. S. Gershon A. S. (2019). Cardiovascular outcomes and all-cause mortality in patients with obstructive sleep apnea and chronic obstructive pulmonary disease (Overlap syndrome). Ann. Am. Thorac. Soc 16, 71–81. doi: 10.1513/AnnalsATS.201802-136OC 30372124

[B23] Köhnlein T. Windisch W. Köhler D. Drabik A. Geiseler J. Hartl S. . (2014). Non-invasive positive pressure ventilation for the treatment of severe stable chronic obstructive pulmonary disease: a prospective, multicentre, randomised, controlled clinical trial. Lancet Respir. Med. 2, 698–705. doi: 10.1016/S2213-2600(14)70153-5 25066329

[B24] Krachman S. L. Tiwari R. Vega M. E. Yu D. Soler X. Jaffe F. . (2016). Effect of emphysema severity on the apnea-hypopnea index in smokers with obstructive sleep apnea. Ann. Am. Thorac. Soc 13, 1129–1135. doi: 10.1513/AnnalsATS.201511-765OC 27078132 PMC5015748

[B25] Lacasse Y. Sériès F. Corbeil F. Baltzan M. Paradis B. Simão P. . (2020). Randomized trial of nocturnal oxygen in chronic obstructive pulmonary disease. N. Engl. J. Med. 383, 1129–1138. doi: 10.1056/NEJMoa2013219 32937046

[B26] Lacasse Y. Sériès F. Vujovic-Zotovic N. Goldstein R. Bourbeau J. Lecours R. . (2011). Evaluating nocturnal oxygen desaturation in COPD--revised. Respir. Med. 105, 1331–1337. doi: 10.1016/j.rmed.2011.04.003 21561753

[B27] Lewis C. A. Fergusson W. Eaton T. Zeng I. Kolbe J. (2009). Isolated nocturnal desaturation in COPD: prevalence and impact on quality of life and sleep. Thorax 64, 133–138. doi: 10.1136/thx.2007.088930 18390630

[B28] Long-Term Oxygen Treatment Trial Research Group Albert R. K. Au D. H. Blackford A. L. Casaburi R. Cooper J. A. Jr . (2016). A randomized trial of long-term oxygen for COPD with moderate desaturation. N. Engl. J. Med. 375, 1617–1624. doi: 10.1056/NEJMoa1604344 27783918 PMC5216457

[B29] Macrea M. Oczkowski S. Rochwerg B. Branson R. D. Celli B. Coleman J. M. . (2020). Long-term noninvasive ventilation in chronic stable hypercapnic chronic obstructive pulmonary disease. An official american thoracic society clinical practice guideline. Am. J. Respir. Crit. Care Med. 202, e74–e87. doi: 10.1164/rccm.202006-2382ST 32795139 PMC7427384

[B30] Malhotra A. Ayappa I. Ayas N. Collop N. Kirsch D. Mcardle N. . (2021). Metrics of sleep apnea severity: beyond the apnea-hypopnea index. Sleep 44, zsab030. doi: 10.1093/sleep/zsab030 33693939 PMC8271129

[B31] Malhotra A. Schwartz A. R. Schneider H. Owens R. L. DeYoung P. Han M. K. . (2018). Research priorities in pathophysiology for sleep-disordered breathing in patients with chronic obstructive pulmonary disease. An official american thoracic society research statement. Am. J. Respir. Crit. Care Med. 197, 289–299. doi: 10.1164/rccm.201712-2510ST 29388824 PMC6835051

[B32] Marin J. M. Soriano J. B. Carrizo S. J. Boldova A. Celli B. R. (2010). Outcomes in patients with chronic obstructive pulmonary disease and obstructive sleep apnea: the overlap syndrome. Am. J. Respir. Crit. Care Med. 182, 325–331. doi: 10.1164/rccm.200912-1869OC 20378728

[B33] Marin J. M. Soriano J. B. Marin-Oto M. De-Torres J. P. Seijo L. M. Cabrera C. . (2025). Sleep-disordered breathing in patients with chronic obstructive pulmonary disease: prevalence and outcomes. Ann. Am. Thorac. Soc 22, 1227–1235. doi: 10.1513/AnnalsATS.202501-030OC 40208311

[B34] McNicholas W. T. (2009). Chronic obstructive pulmonary disease and obstructive sleep apnea: overlaps in pathophysiology, systemic inflammation, and cardiovascular disease. Am. J. Respir. Crit. Care Med. 180, 692–700. doi: 10.1164/rccm.200903-0347PP 19628778

[B35] McNicholas W. T. (2017). COPD-OSA overlap syndrome: evolving evidence regarding epidemiology, clinical consequences, and management. Chest 152, 1318–1326. doi: 10.1016/j.chest.2017.04.160 28442310

[B36] McNicholas W. T. Verbraecken J. Marin J. M. (2013). Sleep disorders in COPD: the forgotten dimension. Eur. Respir. Rev. 22, 365–375. doi: 10.1183/09059180.00003213 23997063 PMC9487346

[B37] Medical Research Council Working Party (1981). Long term domiciliary oxygen therapy in chronic hypoxic cor pulmonale complicating chronic bronchitis and emphysema. Lancet 1, 681–686. doi: 10.1016/S0140-6736(81)91970-X 6110912

[B38] Mehta V. Vasu T. S. Phillips B. Chung F. (2013). Obstructive sleep apnea and oxygen therapy: a systematic review of the literature and meta-analysis. J. Clin. Sleep Med. 9, 271–279. doi: 10.5664/jcsm.2500 23493498 PMC3578679

[B39] Mokhlesi B. Masa J. F. Brozek J. L. Gurubhagavatula I. Murphy P. B. Piper A. J. . (2019). Evaluation and management of obesity hypoventilation syndrome. An official american thoracic society clinical practice guideline. Am. J. Respir. Crit. Care Med. 200, e6–e24. doi: 10.1164/rccm.201905-1071ST 31368798 PMC6680300

[B40] Murphy P. B. Rehal S. Arbane G. Bourke S. Calverley P. M. A. Crook A. M. . (2017). Effect of home noninvasive ventilation with oxygen therapy vs oxygen therapy alone on hospital readmission or death after an acute COPD exacerbation: A randomized clinical trial. JAMA 317, 2177–2186. doi: 10.1001/jama.2017.4451 28528348 PMC5710342

[B41] National Institute for Health and Care Excellence (2021). Obstructive Sleep Apnoea/Hypopnoea Syndrome and Obesity Hypoventilation Syndrome in Over 16s (London: National Institute for Health and Care Excellence). 34613677

[B42] Netzer N. Eliasson A. H. Netzer C. Kristo D. A. (2001). Overnight pulse oximetry for sleep-disordered breathing in adults: a review. Chest 120, 625–633. doi: 10.1378/chest.120.2.625 11502669

[B43] Nocturnal Oxygen Therapy Trial Group (1980). Continuous or nocturnal oxygen therapy in hypoxemic chronic obstructive lung disease: a clinical trial. Ann. Intern. Med. 93, 391–398. doi: 10.7326/0003-4819-93-3-391 6776858

[B44] O'Donoghue F. J. Catcheside P. G. Ellis E. E. Grunstein R. R. Pierce R. J. Rowland L. S. . (2003). Sleep hypoventilation in hypercapnic chronic obstructive pulmonary disease: prevalence and associated factors. Eur. Respir. J. 21, 977–984. doi: 10.1183/09031936.03.00066802 12797491

[B45] Owens R. L. Malhotra A. (2010). Sleep-disordered breathing and COPD: the overlap syndrome. Respir. Care 55, 1333–1344. doi: 10.4187/respcare.10551333 20875160 PMC3387564

[B46] Petrof B. J. Legaré M. Goldberg P. Milic-Emili J. Gottfried S. B. (1990). Continuous positive airway pressure reduces work of breathing and dyspnea during weaning from mechanical ventilation in severe chronic obstructive pulmonary disease. Am. Rev. Respir. Dis. 141, 281–289. doi: 10.1164/ajrccm/141.2.281 2405757

[B47] Rabec C. Rodenstein D. Leger P. Rouault S. Perrin C. Gonzalez-Bermejo J. (2011). Ventilator modes and settings during non-invasive ventilation: effects on respiratory events and implications for their identification. Thorax 66, 170–178. doi: 10.1136/thx.2010.142661 20947891

[B48] Randerath W. Bassetti C. L. Bonsignore M. R. Farre R. Ferini-Strambi L. Grote L. . (2018). Challenges and perspectives in obstructive sleep apnoea: Report by an ad hoc working group of the Sleep Disordered Breathing Group of the European Respiratory Society and the European Sleep Research Society. Eur. Respir. J. 52, 1702616. doi: 10.1183/13993003.02616-2017 29853491

[B49] Redolfi S. Yumino D. Ruttanaumpawan P. Yau B. Su M. C. Lam J. . (2009). Relationship between overnight rostral fluid shift and Obstructive Sleep Apnea in nonobese men. Am. J. Respir. Crit. Care Med. 179, 241–246. doi: 10.1164/rccm.200807-1076OC 19011149

[B50] Robinson T. D. Freiberg D. B. Regnis J. A. Young I. H. (2000). The role of hypoventilation and ventilation-perfusion redistribution in oxygen-induced hypercapnia during acute exacerbations of chronic obstructive pulmonary disease. Am. J. Respir. Crit. Care Med. 161, 1524–1529. doi: 10.1164/ajrccm.161.5.9904119 10806149

[B51] Rossi A. Aisanov Z. Avdeev S. Di Maria G. Donner C. F. Izquierdo J. L. . (2015). Mechanisms, assessment and therapeutic implications of lung hyperinflation in COPD. Respir. Med. 109, 785–802. doi: 10.1016/j.rmed.2015.03.010 25892293

[B52] Sanchez-Azofra A. Gu W. Masso-Silva J. A. Sanz-Rubio D. Marin-Oto M. Cubero P. . (2023). Inflammation biomarkers in OSA, chronic obstructive pulmonary disease, and chronic obstructive pulmonary disease/OSA overlap syndrome. J. Clin. Sleep Med. 19, 1447–1456. doi: 10.5664/jcsm.10600 37082823 PMC10394367

[B53] Schwab R. J. Badr S. M. Epstein L. J. Gay P. C. Gozal D. Kohler M. . (2013). An official American Thoracic Society statement: continuous positive airway pressure adherence tracking systems. The optimal monitoring strategies and outcome measures in adults. Am. J. Respir. Crit. Care Med. 188, 613–620. doi: 10.1164/rccm.201307-1282ST 23992588 PMC5447296

[B54] Shawon M. S. Perret J. L. Senaratna C. V. Lodge C. Hamilton G. S. Dharmage S. C. (2017). Current evidence on prevalence and clinical outcomes of co-morbid obstructive sleep apnea and chronic obstructive pulmonary disease: A systematic review. Sleep Med. Rev. 32, 58–68. doi: 10.1016/j.smrv.2016.02.007 28169105

[B55] Silva Júnior J. L. R. Conde M. B. Corrêa K. S. Rabahi H. Rocha A. A. Rabahi M. F. (2017). Sleep-disordered breathing in patients with COPD and mild hypoxemia: prevalence and predictive variables. J. Bras. Pneumol 43, 176–182. doi: 10.1590/S1806-37562016000000051 28746527 PMC5687947

[B56] Soler X. Liao S. Y. Marin J. M. Lorenzi-Filho G. Jen R. DeYoung P. . (2017). Age, gender, neck circumference, and Epworth sleepiness scale do not predict obstructive sleep apnea (OSA) in moderate to severe chronic obstructive pulmonary disease (COPD): The challenge to predict OSA in advanced COPD. PLoS One 12, e0177289. doi: 10.1371/journal.pone.0177289 28510598 PMC5433709

[B57] Spruit M. A. Singh S. J. Garvey C. ZuWallack R. Nici L. Rochester C. . (2013). An official American Thoracic Society/European Respiratory Society statement: key concepts and advances in pulmonary rehabilitation. Am. J. Respir. Crit. Care Med. 188, e13–e64. doi: 10.1164/rccm.201309-1634ST 24127811

[B58] StanChina M. L. Welicky L. M. Donat W. Lee D. Corrao W. Malhotra A. (2013). Impact of CPAP use and age on mortality in patients with combined COPD and obstructive sleep apnea: the overlap syndrome. J. Clin. Sleep Med. 9, 767–772. doi: 10.5664/jcsm.2916 23946706 PMC3716667

[B59] Sterling K. L. Pépin J. L. Linde-Zwirble W. Chen J. Benjafield A. V. Cistulli P. A. . (2022). Impact of positive airway pressure therapy adherence on outcomes in patients with obstructive sleep apnea and chronic obstructive pulmonary disease. Am. J. Respir. Crit. Care Med. 206, 197–205. doi: 10.1164/rccm.202109-2035OC 35436176 PMC9887426

[B60] Storre J. H. Steurer B. Kabitz H. J. Dreher M. Windisch W. (2007). Transcutaneous PCO2 monitoring during initiation of noninvasive ventilation. Chest 132, 1810–1816. doi: 10.1378/chest.07-1173 18079217

[B61] Voulgaris A. Gogali A. Kostikas K. Steiropoulos P. (2026). Management of COPD-OSA overlap syndrome beyond standard care. COPD 23, 2599583. doi: 10.1080/15412555.2025.2599583 41504509

[B62] Wei X. Zhang X. Wang S. Tong X. Wu C. Hu S. . (2026). Diagnosis and management of overlap syndrome (COPD-OSA): evidence, challenges and prospects. Nat. Sci. Sleep 18, 560579. doi: 10.2147/NSS.S560579 41877850 PMC13006181

[B63] Weitzenblum E. Chaouat A. (2004). Sleep and chronic obstructive pulmonary disease. Sleep Med. Rev. 8, 281–294. doi: 10.1016/j.smrv.2004.03.006 15233956

[B64] Yumino D. Redolfi S. Ruttanaumpawan P. Su M. C. Smith S. Newton G. E. . (2010). Nocturnal rostral fluid shift: a unifying concept for the pathogenesis of obstructive and central sleep apnea in men with heart failure. Circulation 121, 1598–1605. doi: 10.1161/CIRCULATIONAHA.109.902452 20351237

