# Supplementary material for: Beyond the apnea–hypopnea index in COPD–OSA overlap syndrome: a mechanism-informed framework for nocturnal oxygenation and ventilation
Source: Front Physiol. 2026 Aug 13;17:1889633. doi: 10.3389/fphys.2026.1889633 (PMC13520669; doi:10.3389/fphys.2026.1889633)
Supplement: Supplementary file 1 [file Table1.docx]

Supplementary Material 1

Search strategy, literature selection, evidence classification, and SANRA-based checklist

Table S1. Search metadata and review scope.

| Database | PubMed/MEDLINE |
| --- | --- |
| Initial search coverage | Database inception to 27 April 2026 |
| Top-up search | 20 May 2026 |
| Language/population focus | English-language adult human literature |
| Review type | Targeted narrative review supporting transparent source identification and mechanism-oriented conceptual and clinical synthesis. |
| Search and source verification | The initial database search was performed by XZ. Key guideline statements, pivotal overlap-syndrome studies, and management-supporting references were cross-checked against source records during manuscript preparation. |

This supplement documents the search strategy, source-identification process, evidence-selection approach, and narrative-review self-assessment used for the targeted review. The review was not designed to generate a PRISMA flow diagram, systematic-review eligibility counts, duplicate screening, pooled estimates, or formal certainty grading. Records were selected purposively according to relevance to four prespecified interpretive domains and were synthesized by physiological mechanism and evidence source rather than counted toward an eligibility flow.

# 1. PubMed/MEDLINE search syntax

The following core searches were run separately. Results were supplemented by guideline sources and reference-list checking. Searches combined Medical Subject Headings and title/abstract terms and were used to identify direct overlap-syndrome evidence together with indirect COPD or OSA evidence when overlap-specific evidence was unavailable.

## Core overlap-syndrome search

(("Pulmonary Disease, Chronic Obstructive"[Mesh] OR COPD[tiab] OR "chronic obstructive pulmonary disease"[tiab]) AND ("Sleep Apnea, Obstructive"[Mesh] OR "obstructive sleep apnea"[tiab] OR OSA[tiab] OR OSAHS[tiab]) AND ("overlap syndrome"[tiab] OR overlap[tiab] OR COPD-OSA[tiab] OR COPD OSA[tiab] OR COPD-OSAHS[tiab])) AND (adult[MeSH Terms] OR adult*[tiab] OR aged[MeSH Terms])

## Nocturnal hypoxemia and desaturation metrics

(("Pulmonary Disease, Chronic Obstructive"[Mesh] OR COPD[tiab] OR "chronic obstructive pulmonary disease"[tiab] OR "overlap syndrome"[tiab]) AND ("nocturnal hypoxemia"[tiab] OR "nocturnal hypoxaemia"[tiab] OR "nocturnal desaturation"[tiab] OR "oxygen desaturation index"[tiab] OR ODI[tiab] OR T90[tiab] OR T88[tiab] OR "mean SpO2"[tiab] OR "minimum SpO2"[tiab] OR "hypoxic burden"[tiab] OR "hypoxemic burden"[tiab])) AND (adult[MeSH Terms] OR adult*[tiab] OR aged[MeSH Terms])

## Hypoventilation, hypercapnia, and carbon dioxide monitoring

(("Pulmonary Disease, Chronic Obstructive"[Mesh] OR COPD[tiab] OR "overlap syndrome"[tiab]) AND (hypoventilation[tiab] OR hypercapnia[tiab] OR "carbon dioxide"[tiab] OR CO2[tiab] OR PaCO2[tiab] OR bicarbonate[tiab] OR "transcutaneous carbon dioxide"[tiab] OR TcCO2[tiab] OR "end tidal carbon dioxide"[tiab] OR ETCO2[tiab])) AND (adult[MeSH Terms] OR adult*[tiab] OR aged[MeSH Terms])

## Positive airway pressure, noninvasive ventilation, and oxygen therapy

(("Pulmonary Disease, Chronic Obstructive"[Mesh] OR COPD[tiab] OR "overlap syndrome"[tiab]) AND ("Continuous Positive Airway Pressure"[Mesh] OR CPAP[tiab] OR "positive airway pressure"[tiab] OR PAP[tiab] OR "Noninvasive Ventilation"[Mesh] OR NIV[tiab] OR "non-invasive ventilation"[tiab] OR "noninvasive ventilation"[tiab] OR "oxygen therapy"[tiab] OR "home oxygen"[tiab] OR LTOT[tiab] OR "long-term oxygen therapy"[tiab])) AND (adult[MeSH Terms] OR adult*[tiab] OR aged[MeSH Terms])

## Obesity-related ventilatory loading and differential diagnosis of obesity hypoventilation syndrome

(("Obesity Hypoventilation Syndrome"[Mesh] OR "obesity hypoventilation syndrome"[tiab] OR OHS[tiab]) AND (COPD[tiab] OR "chronic obstructive pulmonary disease"[tiab] OR "obstructive sleep apnea"[tiab] OR OSA[tiab] OR overlap[tiab])) AND (adult[MeSH Terms] OR adult*[tiab] OR aged[MeSH Terms])

# 2. Guideline and hand-searched sources

- Global Initiative for Chronic Obstructive Lung Disease. Global Strategy for Prevention, Diagnosis and Management of COPD: 2026 Report.
- American Academy of Sleep Medicine diagnostic testing guidance: Kapur et al., 2017, Clinical Practice Guideline for Diagnostic Testing for Adult Obstructive Sleep Apnea.
- American Academy of Sleep Medicine respiratory-event and hypoventilation scoring guidance: The AASM Manual for the Scoring of Sleep and Associated Events, Version 3 (2023).
- National Institute for Health and Care Excellence guideline NG202 (2021): obstructive sleep apnoea/hypopnoea syndrome, obesity hypoventilation syndrome, and COPD-OSAHS overlap syndrome in people over 16 years.
- American Thoracic Society guideline on obesity hypoventilation syndrome (Mokhlesi et al., 2019).
- American Thoracic Society guideline on long-term noninvasive ventilation in chronic stable hypercapnic COPD (Macrea et al., 2020) and European Respiratory Society guideline on long-term home noninvasive ventilation for COPD (Ergan et al., 2019).
- American Thoracic Society clinical practice guideline on home oxygen therapy for adults with chronic lung disease (Jacobs et al., 2020), together with reference lists of key overlap-syndrome, COPD, OSA, oxygen-therapy, noninvasive-ventilation, and sleep-related ventilation studies.

# 3. Narrative selection, verification, and synthesis priorities

1. Guidelines defining diagnostic or treatment boundaries.
2. Overlap syndrome-specific cohorts and real-world studies reporting hypoxemia, positive airway pressure adherence, exacerbations, hospitalization, cardiovascular outcomes, or mortality.
3. Chronic obstructive pulmonary disease and obstructive sleep apnea trials used when overlap syndrome-specific evidence was unavailable, with evidence indirectness stated in the manuscript.
4. Physiologic studies clarifying sleep-related gas exchange, upper-airway obstruction, lung volume, hypoventilation, CO₂ retention, or oxygen-related hypercapnia.
5. Metric studies on apnea-hypopnea index, oxygen desaturation index, T90/T88, mean SpO₂, nadir SpO₂, hypoxic burden, and transcutaneous CO₂ when relevant to clinical interpretation.

The initial search was performed by XZ. Key guideline statements, pivotal overlap-syndrome studies, and management-supporting sources were cross-checked during manuscript preparation. Evidence was organized into four prespecified domains: objective confirmation of COPD and OSA; nocturnal oxygenation burden; ventilatory adequacy and carbon dioxide retention; and responses to positive airway pressure, noninvasive ventilation, oxygen, and COPD-directed treatment. No PRISMA record counts, duplicate screening, formal risk-of-bias assessment, or certainty grading were performed because the review was conceptual and mechanism-oriented rather than systematic.

# 4. Literature not prioritized

Pediatric studies, case reports, non-English reports, and studies without clear relevance to adult sleep-related oxygenation, ventilation, or management were not prioritized. COPD-only or OSA-only studies were retained selectively when they supplied guideline, randomized, or physiological evidence that was unavailable in overlap-syndrome-specific cohorts; this indirectness is stated in the manuscript.

# 5. Evidence-use statement

Because the review was conceptual and mechanism-oriented rather than systematic, management statements in the manuscript are classified by evidence basis: guideline-supported recommendations, direct overlap-syndrome observational evidence, indirect COPD or OSA trial evidence, and physiological inference. This classification is intended to prevent extrapolated statements from being interpreted as overlap-syndrome-specific guideline-level recommendations.

# 6. SANRA-based checklist for this narrative review

The SANRA domains were used as a structured self-assessment aid to improve transparency and balance; no formal numerical quality score was assigned.

Table S2. SANRA-based checklist for this narrative review.

| SANRA item | How the item was addressed |
| --- | --- |
| Justification of the article's importance for the readership | The introduction defines the clinical problem of apnea-hypopnea index/oxygenation-ventilation discordance in confirmed overlap syndrome and explains why isolated nocturnal hypoxemia should be separated from overlap syndrome. |
| Statement of concrete aims or formulation of questions | The review states a narrow post-diagnostic aim: interpreting nocturnal oxygenation and ventilation once chronic obstructive pulmonary disease and obstructive sleep apnea are both objectively confirmed. |
| Description of the literature search | Search date, database, core PubMed syntax, guideline sources, hand-searching, language/population focus, selection priorities, and non-systematic limitations are documented in this Supplementary Material and summarized in the manuscript. |
| Referencing | The manuscript uses author-date references and cites guidelines, overlap syndrome-specific cohorts, chronic obstructive pulmonary disease/obstructive sleep apnea trials, physiologic studies, and metric studies according to the clinical claim being supported. |
| Scientific reasoning | The synthesis separates diagnostic boundaries, baseline mechanisms, post-treatment residual hypoxemia, and the evidence basis for continuous positive airway pressure, noninvasive ventilation, and oxygen therapy. |
| Appropriate presentation of data | The manuscript avoids pooled estimates and formal certainty ratings, presents tables and figures as interpretive tools rather than treatment algorithms, and states where evidence is direct, indirect, guideline-supported, or physiologic. |
